# Visual-perceptive assessment of glottic characteristics of vocal nodules by means of high-speed videoendoscopy

**DOI:** 10.1016/j.bjorl.2023.05.002

**Published:** 2023-05-18

**Authors:** Gustavo Polacow Korn, Ana Cristina Côrtes Gama, Ualisson Nogueira do Nascimento

**Affiliations:** a Universidade Federal de São Paulo, São Paulo, SP, Brazil; b Universidade Federal de Minas Gerais (UFMG), Belo Horizonte, MG, Brazil

**Keywords:** Vocal cords, Vocal cord dysfunction, Laryngoscopy, Endoscopy

## Abstract

**Objective:**

Visual-perceptive assessment of glottic characteristics of vocal nodules by means of high-speed videoendoscopy.

**Methods:**

Descriptive observational research with convenience sampling of five laryngeal videos of women with an average age of 25 years. The diagnosis of vocal nodules was defined by two otolaryngologists, with 100% intra-rater agreement and 53.40% inter-rater agreement and five otolaryngologists as judge assessed the laryngeal videos based on an adapted protocol. The statistical analysis calculated measures of central tendency and dispersion, as well as percentage. The AC1 coefficient was used for agreement analysis.

**Results:**

In high-speed videoendoscopy imaging, vocal nodules are characterized by amplitude of the mucosal wave and muco-undulatory movement with magnitude between 50% and 60%. Non-vibrating segments of vocal folds are scarce, and the glottal cycle does not show a predominant phase, it is symmetric and periodic. Glottal closure is characterized by the presence of a mid-posterior triangular chink (double chink or isolated mid-posterior triangular chink), without movement of supraglottic laryngeal structures, with irregular contour of the free edge of vocal folds, which are vertically on-plane.

**Conclusion:**

Vocal nodules present mid-posterior triangular chink and irregular free edge contour. Amplitude and mucosal wave were partially reduced.

**Level of evidence:**

Level 4 (Case-series).

## Highlights

• Vocal nodules present mid-posterior triangular chink.
• Vocal nodules present irregular free edge contour.
• In vocal nodules glottal cycle is symmetric and periodic.

## Introduction

Vocal Folds Vibration Pattern (VFVP) observations during phonation offer the most precise data for functional laryngeal assessment.^1^, ^2^

For clinical purposes, Videolaryngostroboscopy (VLS) and high-speed Videoendoscopy (HSV) are methods that record the movement of the VFVP and provide information about the laryngeal structures and tissue mechanics.^3^, ^4^ In terms of quantitative and qualitative assessment, both tools have inherent limitations.^5^, ^6^

VFVP functions and anatomical structures of the larynx are evaluated using visual-perceptual protocols in the clinical setting.^4^

Although VLS has been used clinically for decades with several protocols,^3^, ^4^, ^7^, ^8^, ^9^ criteria for measuring parameters or reliability are still lacking, since existing protocols vary in the combination of parameters.^4^ Furthermore, the protocols lack a clear objective definition of parameters, lack structured training for raters, and observer bias.^4^ Additionally, a protocol using HSV instead of VLS shows more accuracy.^8^

Due to its increased speed above the frequency of vibration of VFVP, HSV records the movement of VFVP cycle-by-cycle and shows the images to the human eye significantly more slowly.^5^ As a result of these characteristics, HSV could be used to assess laryngeal function.^5^

Due to these characteristics, HSV may be the most accurate method for functional assessment of voice disorders.^2^, ^10^ Clinical implementation of HSV may lead to significant improvements in the diagnosis and treatment of vocal fold pathologies.^2^, ^11^, ^12^ Differential diagnosis and defining an effective treatment plan are part of this process.^2^, ^11^, ^12^

Vocal nodules show the presence of bilateral lesion in laryngoscopy, and symmetry of position and usually of size (asymmetry regarding size may occur), symmetric mucosal wave in phase and asymmetric in amplitude, and glottic coaptation characterized by the presence of mid-posterior triangular chink and in some cases also anterior fusiform chink (configuring the double chink).^13^

While vocal fold nodules are one of the most common lesions seen in laryngology clinics,^14^, ^15^ most studies analyze such lesions based on VLS. The literature describes research on quantifying vocal fold nodules through HSV,^11^, ^16^ however, qualitative assessment of the laryngeal image of these lesions obtained through HSV is uncommon.

Understanding the functional behavior of the glottal cycle of vocal fold nodules is important to describe the main clinical signs of these benign lesions, which are so common in laryngeal assessment. The goal of the current study is the visual-perceptive assessment of glottic characteristics of vocal nodules through HSV.

## Methods

### Database

This descriptive observational study was approved by the Research Ethics Committee of the Federal University of Minas Gerais-UFMG, MG, Brazil, under protocol number 1,126,016. An initial convenience sample was formed with 30 laryngeal videos obtained through HSV at the Observatory of Functional Health in Speech and Language Pathology of the Federal University of Minas Gerais (OSF/UFMG). Inclusion criteria consisted of adults within 18–55 age range, and the presence of vocal complaints. They were based on analysis of self-perception of vocal quality (refer having/not having a good or very good voice) and analysis of the presence/absence of vocal symptoms (fatigue and/or discomfort). Presence of vocal complaints was observed through the negative self-perception of vocal quality and the presence of vocal symptoms. Exclusion criteria consisted of patients who reported tobacco use, history of cervical surgery, pregnancy or being in the menstrual or premenstrual period, endocrine disorders, and the use of any systemic medication, as well as signs of laryngopharyngeal reflux, laryngeal disorders of neurological origin and gag reflex during the HSV exam.

Larynx examination consisted of HSV assessment made up by 2000 frames per second, using a 70° rigid laryngoscope with a 300 W xenon light (KayPentax®) with 8-bit RGB color mode high-speed videolaryngoscopy system (model 9710) with 512 × 512 image resolution performed by two otolaryngologists. All participants were instructed to utter the vowel sounds /i/ and /ε/. To standardize loudness and pitch, a speech-language pathologist monitored all recordings.

### First stage

Four otolaryngologists with more than 20 years’ experience in laryngeal assessment were invited to classify the HSV videos and diagnose them as vocal nodules. Using the Zoom® platform, the HSV videos were displayed at 25 frames per second individually to each rater via video call, and the assessment was conducted independently.

Raters were initially shown a composite of images of the glottal cycle extracted from the HSV video. Then the video per se was shown with the possibility of rewatching it up to three times, if necessary. At the end of the HSV video presentation, raters classified vocal nodules in three categories: ‘yes’, ‘no’, or ‘likely’. Twenty percent of the sample was replicated; therefore, six replicas were added to the 30 videos for intra and inter-rater agreement analysis, totaling 36 laryngeal videos.

Among the four raters, the response of the two judges who presented an almost perfect intra-rater agreement (100%) was chosen.^17^ The inter-rater agreement of these two judges was 53.40%, classified as moderate.^17^

The diagnosis of vocal nodules was based on the scores of the two raters. Subjects that obtained two ‘Yes’ responses, or a ‘Yes’ and a ‘Likely' response were considered. A laryngologist and study researcher did the final check of responses. Therefore, at the end of this first stage, out of the 30 videos selected, a final sample of five laryngeal videos of HSV was defined, characterized as women within the 20–29 age range, with an average of 25 years (DP = 3.32) and diagnosis of the presence of vocal nodules (Table 1).

**Table 1:** Distribution of HSV laryngeal videos selected with the diagnosis of vocal fold nodules by raters in the first stage.

Cases	Age	Gender	Rater 1	Rater 2
Case 1	20	Female	Yes	Yes
Case 2	24	Female	Yes	Yes
Case 3	29	Female	Yes	Yes
Case 4	26	Female	Yes	Likely
Case 5	26	Female	Yes	Yes

‘Yes’, Positive for vocal fold nodules; ‘Likely’, Probable vocal fold nodules.

### Second stage

Five judge-raters, laryngologists with average age of 65.4 years (±6.54; between 58 and 76 years) and average time of experience in laryngeal assessment of 39 years (±7.87; between 32 and 52 years) analyzed five HSV video exams of vocal nodules through an adapted protocol^4^ of assessment of vocal fold vibration patterns for HSV.

**Figure 1:**
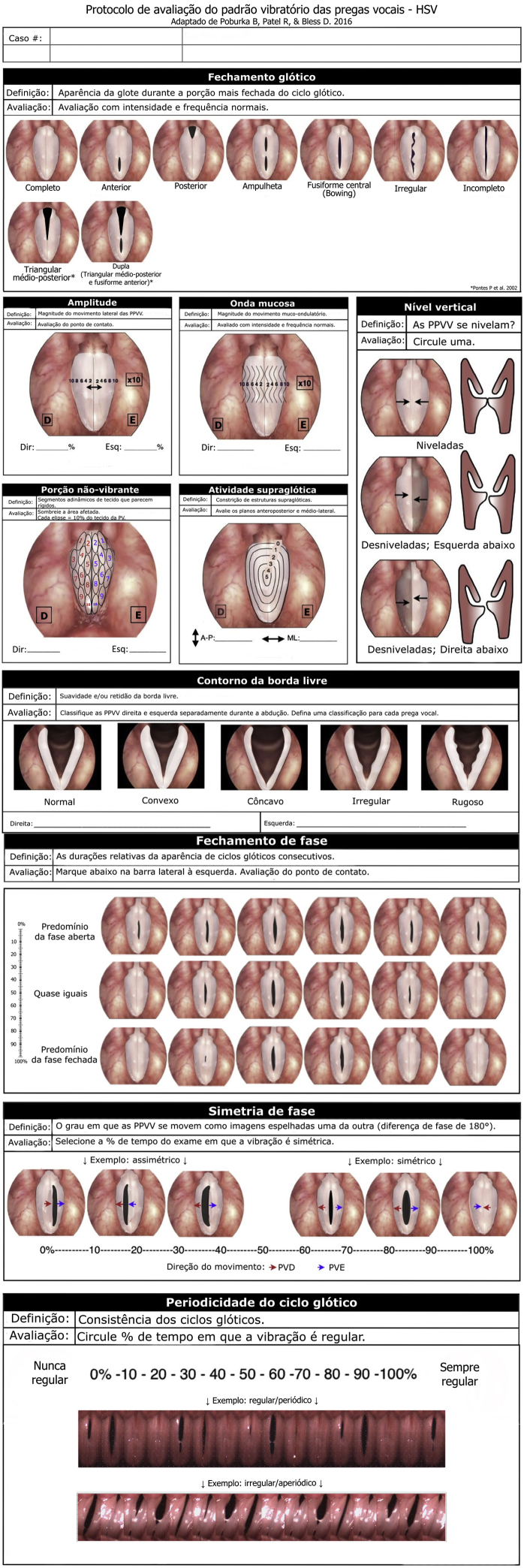
Assessment protocol of vocal folds vibration pattern for high-speed videolaryngoscopy, adapted from the VALI protocol^4^ an translated into Brazilian Portuguese.

An adapted version of the Voice-Vibratory Assessment with Laryngeal Imaging (VALI) Form^4^ was used for the assessment of HSV videos (Fig. 1).

To assess glottal closure, besides seven illustrations,^4^ two more illustrations to assess the types of glottal closure pattern related to the scope of the present study were created by the authors and added to the protocol. Namely, the mid-posterior triangular chink, which results from hyperkinesis and precedes vocal nodules; and the double chink with insufficient coaptation in two regions (mid-posterior triangular chink and anterior fusiform chink), and they are usually associated to the presence of vocal nodules.^13^

**Figure 2:**
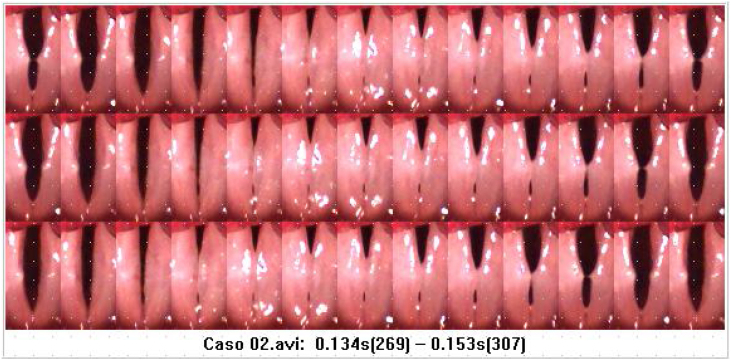
Glottal cycle image composite of the high-speed videolaryngoscopy video, referring to Case 2.

**Figure 3:**
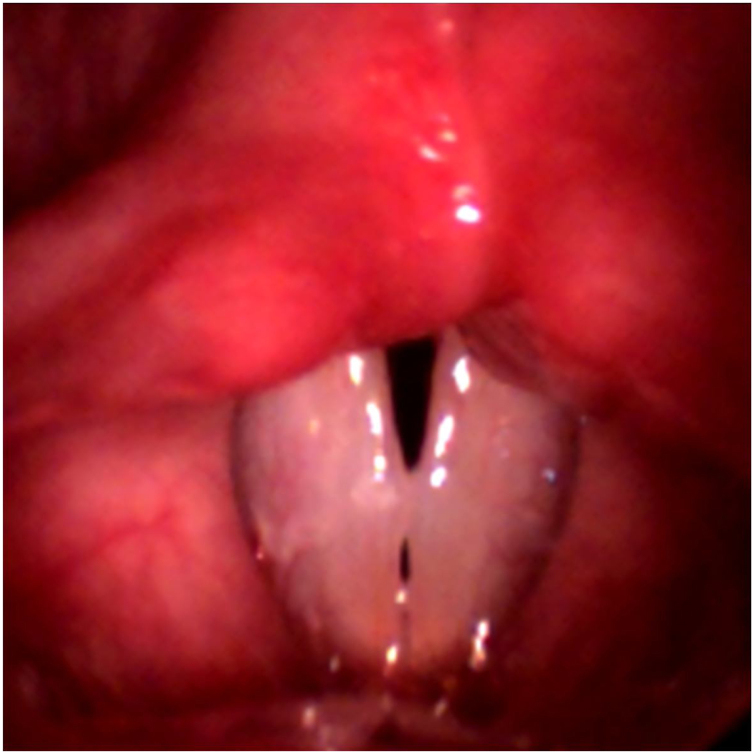
Frame of the high-speed videolaryngoscopy video referring to Case 2. Presence of bilateral lesion between the anterior and middle third is observed; the position is symmetric, with double chink (mid-posterior triangular and anterior fusiform).

To assess cases, one of the researchers got together with each judge-rater and shared the computer screen that allowed the display of a window with HSV video reproductions, another one with composites of glottal cycle images extracted from the HSV (Figure 2, Figure 3), and a third one with the assessment protocol.

The HSV videos were displayed at 50 frames per second. In case the judge-rater needed more reproductions, the video was replayed, and the number of times was written down. The video was reproduced over again at every protocol parameter. The researcher took notes of judge-raters’ responses.

### Statistical analysis

Statistical analysis of data was made using the MINITAB program, version 17. For quantitative variables, a descriptive analysis was made, with measures of central tendency and dispersion. For categorical variables, occurrence frequency assessment was used by means of percentage calculation. The statistical test of first-order Agreement Coefficient (AC1) was used in the first stage of research to assess intra and inter-rater agreement through the R statistical program, version 3.3.1. The agreement level was analyzed considering values of the literature.^17^

## Results

Table 2 shows the distribution in percentage of raters’ responses to qualitative variables (categorical) of the assessment protocol. Vocal nodules are characterized by a glottal cycle with the presence of a mid-posterior triangular chink (double chink or isolated mid-posterior triangular chink), without movement of supraglottic structures, with irregular contour of the free margin of both VFVPs, which are vertically leveled.

**Table 2:** Distribution of qualitative variables of parameters for the adapted protocol of assessment of vocal folds vibration pattern for high-speed videolaryngoscopy for imaging exams categorized as vocal nodules.

Qualitative variables	*n* (%)
*Glottal closure*	
Double chink	11 (44)
Mid-posterior triangular chink	5 (20)
Hourglass chink	5 (20)
Posterior chink	3 (12)
Irregular chink	1 (4)
Complete closure	0 (0)
Anterior chink	0 (0)
Spindle chink	0 (0)
Incomplete closure	0 (0)
*Anteroposterior supraglottic activity*	
Level 0	12 (48)
Level 1	7 (28)
Level 2	5 (20)
Level 3	1 (4)
*Mediolateral supraglottic activity*	
Level 0	19 (76)
Level 1	6 (24)
Level 2	0 (0)
Level 3	0 (0)
*Free edge contour of the right vocal fold*	
Irregular	18 (72)
Normal	3 (12)
Rough	2 (8)
Convex	2 (8)
Concave	0 (0)
Free edge contour of the left vocal fold	
Irregular	18 (72)
Normal	4 (16)
Rough	2 (8)
Convex	1 (4)
Concave	0 (0)
*Vertical level*	
On-plane	23 (92)
Off-plane, left lower	1 (4)
Off-plane, right lower	0 (0)
Non assessable	1 (4)

Results in Table 3 show the distribution in percentage of judge-raters’ responses to quantitative variables (numerical) of the assessment protocol. Amplitude of the mucosal wave and the muco-ondulatory movement of both vocal folds presented a magnitude between 50% and 60%. Non-vibrating segments of both vocal folds are scarce, and the glottal cycle does not show a predominant phase.

**Table 3:** Distribution of quantitative variables of parameters for the adapted protocol of assessment of vocal folds vibratory pattern for high-speed videolaryngoscopy for imaging exams categorized as vocal nodules.

Quantitative variables	Minimum	Maximum	Average	Standard-deviation
Amplitude of the right vocal fold	20	100	61.6	27.03
Amplitude of the left vocal fold	20	100	52.8	26.38
Mucosal wave of the right focal fold	20	100	65.6	23.47
Mucosal wave of the left vocal fold	20	100	56.0	23.09
Nonvibrating portions of the right focal fold	0	80	9.6	16.95
Nonvibrating portions of the left focal fold	0	80	18.4	24.95
Phase closure	20	80	45.2	14.75
Phase symmetry	0	100	69.6	34.58
Periodicity of the glottal cycle	40	100	92.4	14.80

## Discussion

Twenty years ago, Pontes et al.^13^ described vocal nodules as bilateral lesions, symmetric in position but not necessarily in relation to size, with the presence of a mid-posterior triangular chink and in some cases the presence of an anterior fusiform chink, configuring a double chink. More common in young women and children (in this case, more common in male children).

In the current study, those two chinks were categorized in 66% of the cases and in the remaining cases posterior, irregular or hourglass chinks were classified. Problems related to qualitative assessment of larynx exams lie in the lack of standardization for intra and inter-institutional comparisons, as well as intra and inter-rater evaluations.^4^ Considering that the qualitative assessment of the laryngoscopy video is not objective, we may assume that the difference between a posterior chink and a mid-posterior triangular chink may create inconsistency in the assessment,^4^ even in the evaluation of laryngologists with extensive experience.

**Figure 4:**
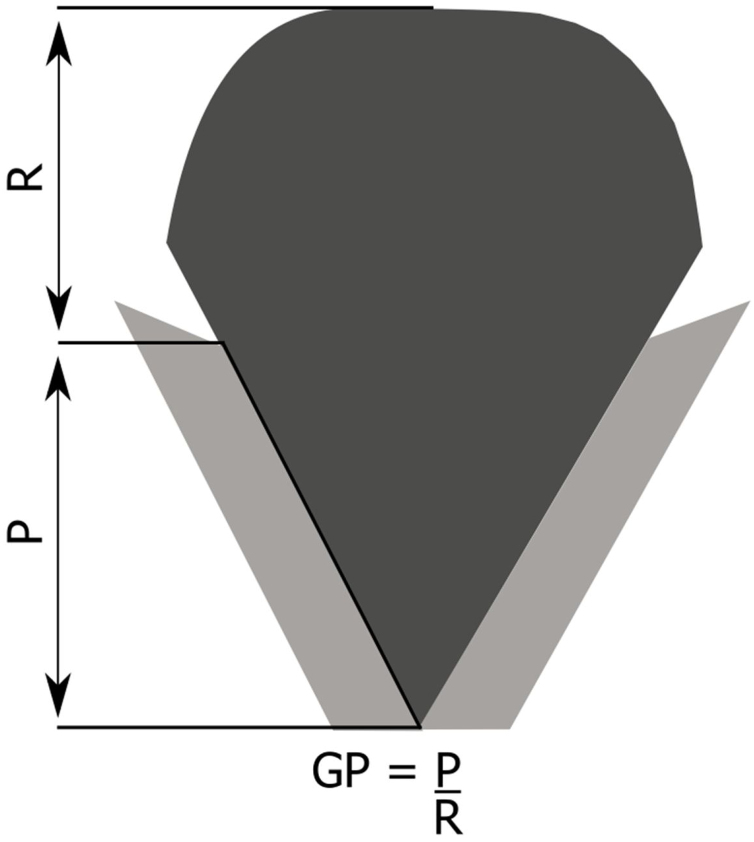
Schematic representation of glottic proportion. P, Phonatory region; R, Respiratory region; GP, Glottic proportion. Figure adapted from Pontes et al.^13^

Glottic Proportion (GP)^18^ is defined as the relation between the anteroposterior dimension of the Phonatory region (P) and the Respiratory Region (R), and the limit between these regions is the projection of arytenoid vocal processes, which show differences regarding gender and age, as observed in Fig. 4.

Lower values of GP^18^ are found in the female gender compared to males (and in children), representing a predisposing factor in the genesis of vocal nodules. For this reason, the female larynx is more predisposed to posterior chink, and possibly to mid-posterior chink.^18^ The shape of the mid-posterior triangular chink causes contact energy between the VFVPs, between the anterior and middle third, and in this situation, the presence of abuse or vocal misuse may result in vocal nodules.

The presence of muscle tension, especially non-relaxation of the posterior cricoarytenoid muscle may favor the presence of mid-posterior triangular chink, which has a larger friction area in the transition region of the anterior and middle third, resulting in the formation of vocal nodules. The size of this area may hinder the closure of the anterior third of the glottis, also resulting in anterior fusiform chink, and then double chink.^18^

Characterizing the type of glottic chink is extremely important for the differential diagnosis of other situations such as the vocal fold cyst, whose presence may occur regardless of the use of voice, resulting in an hourglass chink, which is different from the chink of vocal nodules.

Even though this concept has been used in Brazil for 20 years,^13^ it is little used internationally, and in literature, the description of vocal fold nodules could be a little more detailed.

In 2012, a renowned group of laryngologists with extensive experience published the article “A Nomenclature Paradigm for Benign Midmembranous Vocal Fold Lesions”,^19^ in which vocal fold nodules were bilateral, there was no need for complete symmetry, mucosal vibration properties were normal, or with a slight impairment. Results of this research suggest that the presence of a mid-posterior triangular chink, in isolated form or double chink should be included as a parameter to classify vocal nodules.

Regarding other laryngeal findings, the research revealed low frequency of anteroposterior and mediolateral supraglottic activity in women with vocal nodules. Literature has shown that the supraglottic activity is a frequent finding in the presence of vocal nodules^20^, ^21^, ^22^ (around 60%–70%). However, this laryngeal parameter may also be observed in individuals with normal larynx.^22^, ^23^ In situations of an altered larynx, the prevalence of supraglottic activity is higher in individuals with functional dysphonia,^21^, ^24^ and in individuals with neurological disorders.^21^, ^23^ The reduced sample of the current research is a limitation for a more accurate presentation regarding the mediolateral and anteroposterior supraglottic activity of the HSV videos analyzed. Increasing the sample for a future study, as well as analyzing quantitative data of supraglottic activity via HSV may provide further clarification of the supraglottic activity in the presence of vocal nodules.

Research findings revealed higher frequency of irregular contour of the free edge of both vocal folds in the presence of vocal nodules, corroborating literature findings of non-normality of the free margin in this type of benign lesion of the larynx.^25^ HSV videos used in the research were short and recorded vocal folds glottal cycle, in other words, videos were not recorded during breathing or with vocal folds in total abduction, ideal situations for the assessment of the free edge contour. However, even with such limitation, analysis of the HSV videos allowed the assessment of this parameter.

Research data revealed that 92% of vocal folds were vertically leveled. This finding is expected, as off-plane is not found in laryngeal lesions such as vocal nodules.^8^ The vertical level off-plane is more frequent in vocal fold atrophy and paresis.^8^

Regarding the vibration amplitude and mucosal wave parameters of vocal folds, we observed values between 50% and 60%. Since vocal nodules are lesions in the superficial layer of lamina propria, they do not cause great repercussion for the vocal folds vibration amplitude.^20^ Literature also shows that the mucosal wave is present in vocal nodules.^20^

The current research showed low occurrence of VFVP nonvibrating portions. Findings of immobility or adynamic segments in the membranous portion of vocal folds is related to lesions where there is absence or mucosal wave stiffness and reduced amplitude such as in carcinoma, papilloma, and vocal fold scars.^3^

Parameters of phase closure, phase symmetry and periodicity of the glottal cycle are better assessed in HSV owing to its ability to identify real cycle-to-cycle changes.^8^ In the current research, there was no predominant phase, there was vocal folds symmetry and periodic movement, which is consistent with literature that shows that vocal nodules show vocal folds with symmetric vibrations or minimal symmetry reduction.^13^, ^20^, ^26^

Instead of using the term vocal fold nodules, Zhukhovitskaya et al.^26^ used the term *midfold mass.* Authors have observed that these lesions occur more frequently in young women. We agree with Nauhenim and Caroll^27^ that the classification system of VFVP lesions is imperfect and keeps evolving, and that the common use of the term “nodules” to describe most vocal folds benign lesions is coming to an end. We believe that the inclusion of the type of glottic chink may contribute significantly to improve classification.

Lee et al.^28^ have recently described the pattern of recurrence of phonotraumatic lesions, suggesting different mechanisms of injury. They have observed that the term *midfold mass* lesion can be associated to a lesion close to vocal nodules. They are chronic lesions predominant in women, suggesting an accumulation of damages in the larynx susceptible to increased phonotrauma.

In the situation of vocal nodules, the susceptible larynx would be the female larynx, which presents lower values of glottic proportion and is more predisposed to the chink. With phonotrauma, it will generate more friction concentration in the region between the anterior and middle third, instead of being distributed along the phonatory portion, and this accumulation results in lesion.

It is interesting to mention that the genesis of vocal nodules in women has been attributed to hyaluronic acid. However, in two studies with cadavers using the same direct method to quantify hyaluronic acid, it was observed that this acid was present in higher concentrations in women than in men,^29^ and in young women its concentration showed great variability.^30^ Therefore, the concentration of hyaluronic acid may be understood as a protection factor in young women.

Regarding laryngoscopy assessment, we would like to suggest an adaptation of VALI,^4^ so that the types of mid-posterior triangular chink and double chink (mid-posterior triangular and anterior fusiform chink) could be included in the protocol.

Predisposition to incomplete glottal closure was related to another benign lesion of vocal folds, the pseudocyst.^17^ Following the concept of lower glottic proportion^19^ in women, which favors incomplete glottal closure, this lesion is also more frequently found in the female gender.^26^, ^28^ Just like in vocal nodules, predisposition to incomplete closure and the cumulative effect of high vocal demand are found. Sulica et al.^31^ observed in 46 patients that 80.43% of them were vocal performers, suggesting that this population is more susceptible to pseudocyst.

In a study on music genre and phonotraumatic lesions, Childs et al.^32^ have observed that the pseudocyst appears more frequently in female opera singers than in theater or chorus singers, and thus related these lesions with a more specific posture of the larynx during performance. Just like Childs et al.,^32^ our experience suggests that vocal fold nodules derive more from speaking than from singing voice.

Studies on vocal assessment of professionals who use their voice as a working tool show more prevalence of speaking voice professionals, with the presence of vocal nodules among the most recurrent laryngeal lesions.^33^, ^34^, ^35^, ^36^

A study that assessed 1093 laryngeal exams revealed that, among non-neoplastic laryngeal lesions in patients with vocal complaints, the prevalence of vocal fold nodules is high,^37^ and vocal fold nodules were characterized by more presence of double chink, followed by mid-posterior triangular chink.^36^, ^37^

The current study has a few limitations. Videos recorded by the HSV in the current study showed 2000 frames per second. Literature suggests recordings at a speed lower than 4000 frames per second may not be sufficient to record real vibration characteristics of vocal folds and should be interpreted with caution.^2^, ^38^ Parameters such as amplitude, mucosal wave, phase closure, phase symmetry and glottal cycle periodicity may suffer the influence of the number of frames per second in the current study.

In the assessment of videos, we opted for judge-raters with extensive experience in laryngology, with an average experience time of 39 years. The judge-raters did not receive any visual-perceptive training to assess HSV videos and to use the protocol. In the original VALI^4^ study, they inform that the classification tool and rater training are inextricably linked in order to improve the reliability of ratings of laryngeal videos.^4^ Not being familiar with descriptions and images of the protocol and the specific observational training to rate HSV videos may influence results, despite years of experience in laryngology of the judge-raters.

## Conclusion

HSV reveals vocal nodules with a mid-posterior triangular chink (double chink or isolated mid-posterior triangular chink) and irregular free edges of the vocal folds. VFVP is symmetrical and periodic, with partially reduced amplitude and mucosal wave.

## Conflicts of interest

The authors declare no conflicts of interest.
